# Supplementary material for: Risk factors for adverse pregnancy outcomes in Chinese women: a meta-analysis
Source: PeerJ. 2023 Sep 12;11:e15965. doi: 10.7717/peerj.15965 (PMC10503497; doi:10.7717/peerj.15965)
Supplement: Supplemental Information 3 [file peerj-11-15965-s003.docx]

**Systematic Review and/or Meta-Analysis Rationale**

**For systematic reviews / meta-analyses, authors need to provide the following information:**

**The rationale for conducting the systematic review / meta-analysis;**

**The contribution that it makes to knowledge in light of previously published related reports, including other meta-analyses and systematic reviews.**

R: The rationale for conducting the systematic review/meta-analysis: Expected adverse pregnancy consequences comprise preterm labor, macrosomia, neonatal low birth weight, stillbirths, abortions, and placental abnormalities. These outcomes significantly impact the physiological and psychological well-being of both pregnant women and neonates and have garnered worldwide attention. Infants born under unfavorable prenatal circumstances are also susceptible to nerve impairment, respiratory ailments, visual and auditory impairments, delayed development in the future, cognitive disability, and cerebral palsy. Hence, there exists a pressing requirement to mitigate the possibility of unfavorable delivery consequences via regulation of risk determinants for adverse pregnancies or surveillance and intervention in expectant women at high risk. Therefore, we conducted a meta-analysis to investigate the risk factors associated with adverse pregnancy outcomes among Chinese women in recent years. The contribution that it makes to knowledge considering previously published related reports, including other meta-analyses and systematic reviews: The interrelated variables were methodically synthesized, and adverse pregnancy consequences were investigated via meta-analysis to direct prenatal and perinatal healthcare and expedite the consequent formulation of suitable interventions to diminish the incidence of adverse pregnancy outcomes. This study has the following limitations, and as with other systematic reviews, our review is limited by publication bias. Published studies were summarized, and no grey literature or other sources were searched. It is also possible that articles are not collected for the restrictions on searching policies. Since this review is limited to studies in China, articles from other countries are inevitably excluded. Besides, certain factors related to adverse pregnancy may be omitted because few reports on such related factors are not authoritative for meta-analysis. Particular retrospective literature was included, and the quality of the study was uneven.

**Systematic Review Registration**

**Please explain why data analysis began before submission to INPLASY.**

R: Thank you for your instructive suggestions. I submitted the registration on April 26, 2023, and the approval date was April 26, 2023. And starting from April 30, 2023, I began to search and collect information until the end of the collection work on May 15, 2023.
